# Supplementary material for: Patterns of Intron Gain and Loss in Fungi
Source: PLoS Biol. 2004 Nov 30;2(12):e422. doi: 10.1371/journal.pbio.0020422 (PMC532390; doi:10.1371/journal.pbio.0020422)
Supplement: Table S1 — Also available at http://genes.mit.edu/NielsenEtAl/. (4.3 MB ZIP). [file pbio.0020422.st001.zip › NielsenEtAl/html/528.html]

AN3771.1.NCU06750.1.MG02510.1.FG06571.1


```
 CLUSTAL W (1.82) Multiple Sequence Alignments - Introns Inserted


Sequence 1: MG02510.1	213 aa
Sequence 2: FG06571.1	221 aa
Sequence 3: NCU06750.1	205 aa
Sequence 4: AN3771.1	148 aa
Alignment Length: 229 aa
Number Identitical Residues: 71 aa
Alignment Score (without introns) 4317


MG02510.1 	MVYYFTSNVVEPSAYIYVGKDKFEN1EDLIKHGWEEDVW2FHADKLSS~AHIYLRLREGE
NCU06750.1	MVFYFTSTVVSPPAFIYVGKDKFEN1EELIQHG------~WEED--VC2AHIYLRLPEGW
FG06571.1 	MVLYFTSSVVDPSGFIYVGKDKFES~--MVHP------K2FHADKLSS~AHIYLRMKEGQ
AN3771.1  	-------------------------~-------------~--------~-----------
          	                                                            

MG02510.1 	TWDKIPEALVTDLAQLTKANSI~E----------------G1NKKDNVTIIYTPWSNLHK
NCU06750.1	TWENIPEELLTDLGQLTKANSI~E----------------G1NKKDNITIIYTPWANLKK
FG06571.1 	TWDALPEELVMDLAQLTKANSI~EGTRLALLISAPVLTPPG~NKKDNVTIIYTPWSNLKK
AN3771.1  	---------MANHGTTSRKSSW2R---------------TG~NKKDNITVIYTPWSNLMK
          	         : : .  :: .*  .               .* *****:*:*****:** *

MG02510.1 	DGSMAVGQVGFKDQKK0VKRVLVPQRENPIVNRLNKTKVEKHPDLRQEKEDHLKELRKGQ
NCU06750.1	DGSMAVGQVSFKDQKK~VKRILIAQRENAIVNRLNKTKVEKHPDLREEKEQHLKELRKKD
FG06571.1 	DGSMEVGQVGFKDPRK~VKRILVPQRENPIVNRLNKTKVEKKPDLKQERDDRLKELRRRD
AN3771.1  	DGSMATGQVSFHNHKL~VKKVFVAQRENAIVNRLNKTKVEKYPDLRAEKEDYKKMLQREE
          	**** .***.*:: :  **::::.****.************ ***: *:::  * *:: :

MG02510.1 	QAAQQER0KKEEARIARERKEKKYQKDHAYDDIFAEENMEASSNQNRSEDWEDDFM
NCU06750.1	QAAVLAR~KKEEDRLKKERAEKKWQKDHAYDELFAEENMMNSSNQDRDETWEDDFM
FG06571.1 	QAVFLAR0KKEEAKQAQEWKEKKYQKDHAYDDIFTEELMAGSSNQDRDENWEDDFM
AN3771.1  	RKIREEK~KNREKQEKREREQLKWQKDHAYDDLFSEENMEASNNQDRDPNFLDDFM
          	:     : *:.* :  :*  : *:*******::*:** *  *.**:*.  : ****
```
